# Characterization of Bitter Off-Taste Stimuli in Sunflower Press Cake Using the Sensomics Approach

**DOI:** 10.1021/acs.jafc.5c07283

**Published:** 2025-09-04

**Authors:** Lachinkhanim Huseynli, Michael Gigl, Jasmin Müller, Christoph Walser, Oliver Frank, Kristel Vene, Corinna Dawid

**Affiliations:** 1 Department of Chemistry and Biotechnology, 428479 Tallinn University of Technology, Akadeemia tee 15, 12618 Tallinn, Estonia; 2 Junior Research Group Food Processing and Health, ZIEL Institute for Food and Health, 9184 Technical University of Munich, Lise-Meitner-Str. 34, D-85354 Freising, Germany; 3 Chair of Food Chemistry and Molecular Sensory Science, TUM School of Life Sciences, 9184 Technical University of Munich, Lise-Meitner-Str. 34, D-85354 Freising, Germany; 4 Professorship for Chemosensory Food Systems, TUM School of Life Sciences, 9184 Technical University of Munich, Lise-Meitner-Str. 34, D-85354 Freising, Germany; 5 Leibniz Institute for Food Systems Biology at the Technical University of Munich, Lise-Meitner-Str. 34, D-85354 Freising, Germany

**Keywords:** sunflower, bitter taste, off-flavor, fatty acid oxidation products, liquid chromatography−tandem mass spectrometry

## Abstract

The Sensomics approach, including activity-guided fractionation and taste dilution analysis, was employed to identify the key compounds responsible for the bitter off-taste of sunflower press cake. A combination of liquid chromatography–tandem mass spectrometry, liquid chromatography–time-of-flight-mass spectrometry, one-/two-dimensional nuclear magnetic resonance spectroscopy, and dose-overthreshold factor calculation led to the identification of 9,12,13-trihydroxyoctadec-10-enoic acid, 9,10,11-trihydroxyoctadec-12-enoic acid, 11,12,13-trihydroxyoctadec-9-enoic acid, (10*E*,12*E*)-9-hydroxyoctadeca-10,12-dienoic acid, (10*E*,12*Z*)-9-hydroxyoctadeca-10,12-dienoic acid, (9*E*,11*E*)-13-hydroxyoctadeca-9,11-dienoic acid, (9*Z*,11*E*)-13-hydroxyoctadeca-9,11-dienoic acid, (9*Z*,11*E*)-13-oxooctadeca-9,11-dienoic acid, α-linolenic acid, linoleic acid, oleic acid, 2-hydroxyoleic acid, palmitic acid, stearic acid, and novel pinocarveol β-d-apiofuranosyl-(1→6)-β-d-(4-*O*-caffeoyl) glucopyranoside as contributors to the bitterness of sunflower press cake. The findings provide valuable insights into the sensory challenges associated with using sunflower press cake in food applications and offer pathways to enhance its palatability and potential as a sustainable protein alternative to meet future protein demands.

## Graphical abstract

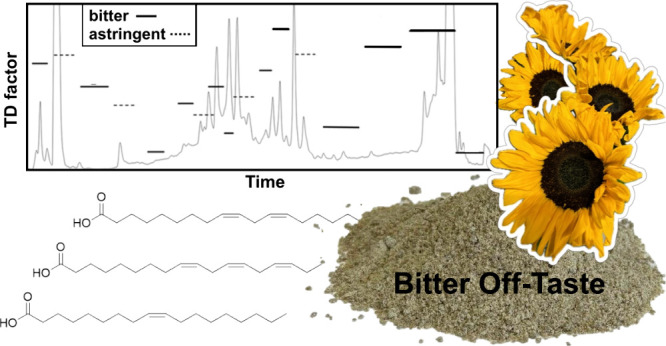

## Introduction

The sunflower (*Helianthus annuus* L.), a member of the Asteraceae family, is cultivated as a crop worldwide for human and livestock consumption.^1,2^ Sunflower is primarily used for oil production, and it is the third most widely cultivated oilseed crop in the world, with a production volume of 57 million tons per year, after soybeans (364 million tons) and rapeseed (71 million tons).^3^ Furthermore, sunflower oil is the fourth most popular vegetable oil worldwide and is often valued for its monounsaturated fatty acid content.^1,4^ Sunflower meal or cake, a byproduct of oil extraction, contains proteins, cellulosic fibers, lignins, phenols, minerals^5^ and is primarily used as fertilizer and animal feed. In 2017, approximately 19 million tons of sunflower press cake was generated worldwide.^6^ The sunflower press cake contains 19.9%–44.9% of protein,^7^ which makes it a valuable potential source to meet the growing global protein demand.^8^ This is especially relevant as food industries increasingly prioritize the development of sustainable protein sources in response to growing population needs.^8^

In light of this, plant-based proteins, used as alternatives to traditional protein sources, are promising not only for their nutritional benefits but also for their potential to contribute to food security. However, the consumer acceptance of these proteins is often compromised by off-flavors, particularly bitter taste.^9–11^ Addressing the factors that contribute to these bitter off-flavors is crucial for optimizing the use of emerging protein sources. Recently, there has been increasing interest in valorizing sunflower meal/cake as a sustainable protein source for food applications.^12–14^ However, despite the high protein content of sunflower meal/cake, research on its sensory properties, particularly taste, remains limited. A recent review has drawn attention to the sensory challenges arising from the limited research on sunflower-derived materials, which may complicate their integration into food applications, suggesting the importance of further exploration of their taste-active compounds.^4^

To understand these sensory challenges more effectively and on a molecular level, the sensomics approach can be used to identify the key taste and off-flavor compounds in food products.^15^ Previous studies have used the sensomics approach to identify taste-active compounds in linseed oil,^16^ hazelnuts,^17^ poppy seeds,^18^ asparagus,^19^ rapeseed protein isolates,^20,21^ and pea protein isolates.^22^ Pickardt et al. (2015) highlighted the presence of a bitter taste in sunflower meal, which may pose a challenge to consumer acceptance.^23^ Therefore, the present study aimed to identify the key compounds contributing to the bitter off-taste of sunflower press cake by means of activity-guided fractionation, taste dilution analysis, and dose-activity calculation.

## Materials and Methods

### Chemicals

The following compounds were obtained commercially: acetonitrile (ACN), methanol (MeOH) (J.T. Baker, Deventer, The Netherlands); acetone, ethyl acetate, *n*-pentane (BDH Prolabo, Briare, France); formic acid (Merck, Darmstadt, Germany), dimethyl sulfoxide-d_6_ (DMSO)–deuterium oxide (D_2_O), linoleic acid, ricinoleic acid, [^13^C_18_]-linoleic acid, α-linolenic acid, hydrochloric acid, anhydrous pyridine, l-cysteine methyl ester hydrochloride, phenylethyl isothiocyanate, d-glucose, d-galactose, d-mannose, d-xylose, d-ribose, d-apiose (Sigma-Aldrich, Steinheim, Germany); isopropyl alcohol (Honeywell, Seelze, Germany); (10*E*,12*E*)-9-hydroxyoctadeca-10,12-dienoic acid, (9*E*,11*E*)-13-hydroxyoctadeca-9,11-dienoic acid, (9*Z*, 11*E*)-13-oxooctadeca-9,11-dienoic acid, 2-hydroxyoleic acid, and 18-hydroxyoleic acid (Larodan AB, Solna, Sweden). The acetonitrile used for high-performance liquid chromatography–tandem mass spectrometry (HPLC–MS/MS) analysis was liquid chromatography–mass spectrometry (LC–MS) grade (Honeywell, Seelze, Germany); acetone, ethyl acetate, and *n*-pentane were distilled before use, and all other solvents were HPLC grade. The water used for chromatographic separation was purified using an Advantage A10 water System (Millipore, Molsheim, France). Bottled water (Evian) was adjusted to pH 5.9 using formic acid and used for sensory analyses. Sunflower press cake was obtained from SUNFLY OU in Estonia.

### Sequential Solvent Extraction

A total of 300 g of sunflower press cake was subjected to solid–liquid extraction with methanol/water (70:30, v/v, 1500 mL) three times by stirring for 30 min at room temperature, followed by centrifugation (4 min, 5000 rpm) and filtration. The filtrates were collected and combined, separated from the solvent by vacuum evaporation at 40 °C, and lyophilized to obtain the MeOH/H_2_O extractables (fraction F1). The residue was extracted further with MeOH (1500 mL, fraction F2), followed by ethyl acetate (1500 mL, fraction F3) and *n*-pentane (1500 mL, fraction F4). The extracted solvent fractions F1–F4 were freeze-dried twice to remove trace amounts of solvents and stored at −20 °C until they were used for a comparative taste profile analysis.

### Fractionation of F1 by Solid-Phase Extraction

For solid-phase extraction fractionation, an aliquot (1 g) of fraction F1 was dissolved in water (40 mL) and sonicated at room temperature (10 min). This solution was separated on a Chromabond C18 end-capped cartridge (45 μm, 70 mL/10 g, Macherey-Nagel, Düren, Germany), which was preconditioned with methanol (2 × 70 mL), followed by water (2 × 70 mL). Elution was performed with water (2 × 70 mL) to obtain fraction F1-1, methanol/water (30:70, v/v, 2 × 70 mL) to obtain fraction F1-2, methanol/water (50:50, v/v, 2 × 70 mL) to obtain fraction F1-3, methanol/water (70:30, v/v, 2 × 70 mL) to obtain fraction F1-4, and methanol (2 × 70 mL) to obtain fraction F1-5. The collected fractions were separated from the solvent by vacuum evaporation at 40 °C, lyophilized twice, and stored at −20 °C until subsequent use for chemical and sensory analyses.

### Separation of Fraction F1-4 by Preparative High-Performance Liquid Chromatography (HPLC)

Fraction F1-4 was dissolved in H_2_O/ACN (80:20, v/v; 320 mg in 6 mL) with ultrasonication at room temperature (10 min). The sample was membrane filtered and injected (300 μL) into a Nucleodur C18 Pyramid column (250 × 21 mm, 5 μm, 110Å, Macherey-Nagel, Düren, Germany) equipped with a guard column of the same type. The separation was performed at a flow rate of 20 mL/min using 0.1% aqueous formic acid as solvent A and acetonitrile as solvent B. The effluent was monitored using a Sedex LT-ELSD detector Model 85 (Sedere, Alfortville, France) at Gain 12. The gradient flow was as follows: 0 min, 15% B; 3 min, 15% B; 28 min, 60% B; 30 min, 100% B; 32 min, 100% B; 35 min, 15% B; 40 min, 15% B. In total, 17 fractions were collected (F1-4-1 to F1-4-17), separated from the solvent (vacuum evaporation at 40 °C), lyophilized twice, and then stored at −20 °C until further use.

### Identification of Fatty Acids and Fatty Acid Oxidation Products as Key Bitter Compounds in Fractions F1-4-15 and F1-4-16

Fractions were subjected to untargeted screening using ultrahigh performance liquid chromatography–time-of-flight–mass spectrometry (UPLC-TOF-MS) to facilitate the identification of compounds within the fractions. To verify these identifications, the samples were analyzed alongside reference compounds using a previously established method.^18,22^ The retention times and mass spectral data obtained in the present analysis were consistent with those of the reference compounds, confirming that the identified compounds aligned with those reported in previous research.^18,22^

### Identification of the Bitter Compound in Fraction F1-4-12

Fraction F1-4-12 was dissolved in H_2_O/ACN (80:20, v/v; 10 mg/mL) and, after membrane filtration, fractionated by semipreparative HPLC using a Luna Phenyl-Hexyl column (250 × 10 mm, 5 μm, 100 Å, Phenomenex, Aschaffenburg, Germany), equipped with a guard column of the same type, with a binary gradient using 0.1% formic acid in H_2_O as solvent A and 0.1% formic acid in ACN as solvent B (flow rate 4.7 mL/min): 0 min, 30% B; 3 min, 30% B; 25 min, 45% B; 28 min, 100% B; 30 min, 100% B; 32 min, 30% B; 35 min, 30% B. For detection DAD detector model MD-2010 Plus (Jasco, Groß-Umstadt, Germany) was used. Fraction F1-4-12-5 contained the bitter target compounds and was collected in multiple HPLC runs, combined, separated from the solvent (vacuum evaporation at 40 °C), and then lyophilized. For further purification fraction F1-4-12-5 was dissolved in ACN/H_2_O (30/70, v/v; 1 mg/mL) and fractionation was performed with an analytical Luna Phenyl-Hexyl column (250 × 4.60 mm, 5 μm, 100 Å, Phenomenex, Aschaffenburg, Germany) with a flow rate of 1 mL/min and using 0.1% formic acid in H_2_O as solvent A and 0.1% formic acid in MeOH as solvent B. DAD detector model MD-2010 Plus (Jasco, Groß-Umstadt, Germany) was used. Separation was performed using the following gradient: 0 min, 50% B; 2 min, 50% B; 29 min, 62% B; 32 min, 100% B; 34 min, 100% B; 36 min, 50% B, 40 min, 50% B. The fraction containing the bitter compound (**15**) was collected in multiple HPLC runs, separated from the solvent, and lyophilized; subsequently, structural analysis was conducted using MS/MS following hydrolysis, TOF-MS, and nuclear magnetic resonance spectroscopy (NMR), as well as sensory threshold analysis.

### Determination of Monosaccharide Constituents via Ultrahigh Performance Liquid Chromatography–Tandem Mass Spectrometry Following Acidic Hydrolysis

Using a previously reported protocol,^24^ the monosaccharide constituents of **15** were determined after acidic hydrolysis. Therefore, the isolated compound **15** (1 mg) was dissolved in a mixture of ACN/H_2_O (3:7, v/v, 100 μL) and treated with hydrochloric acid (6 mol/L, 1 mL). The mixture was heated at 100 °C for 60 min. After heating, the mixture was evaporated to dryness under nitrogen. The resulting residue was resuspended in 2 mL of H_2_O and extracted three times with 2 mL of ethyl acetate. The aqueous layer was then evaporated to dryness to obtain a monosaccharide-containing residue. This residue was dissolved in anhydrous pyridine (100 μL), and a solution of l-cysteine methyl ester hydrochloride (500 μL, 2 mg/mL) was added. The mixture was shaken at 60 °C at 1400 rpm in a thermo shaker (PHMT–PSC24N, Grant Bio, Cambridge, UK) for 60 min. Next, phenylethyl isothiocyanate (50 μL) was added to the solution, and the resulting mixture was shaken again at 60 °C for 60 min. The mixture was dried under a stream of nitrogen, reconstituted in a mixture of ACN/H_2_O (1:1, v/v, 500 μL), and transferred to an autosampler vial; then, an aliquot (1 μL) was subjected to UHPLC-MS/MS analysis. Mass spectrometry was conducted using a QTRAP 6500 mass spectrometer (AB Sciex, Darmstadt, Germany) operated in ESI+ mode. The ion source parameters were as follows: ion spray voltage at 5500 V (ESI^+^), curtain gas at 35 psi, nebulizer gas at 55 psi, heater gas at 65 psi, collision-activated dissociation high, and source temperature 500 °C. The MS system was coupled to a Shimadzu Nexera X2 UHPLC (Shimadzu, Duisburg, Germany). The system consisted of two pumps (LC-30AD), a degasser (DGU-20A5R), an autosampler (SIL-30AC), a column oven (CTO-30A), and a controller (CBM-20A). Data acquisition was performed using Analyst 1.6.3 (AB Sciex, Darmstadt, Germany). For all reference compounds, individual MS/MS parameters were first tuned and optimized on the UHPLC-MS/MS system for each compound after derivatization. After optimizing instrument settings with reference compounds, the derivatized monosaccharides were analyzed using the mass transitions Q1/Q3 of *m*/*z* 461.0/298.1 (DP = 86 V, CE = 17 V, CXP = 6 V) for d-glucose, Q1/Q3 of *m*/*z* 461.1/298.2 (DP = 71 V, CE = 17 V, CXP = 6 V) for d-galactose, Q1/Q3 of *m*/*z* 461.0/298.1 (DP = 71 V, CE = 17 V, CXP = 6 V) for d-mannose, Q1/Q3 of *m*/*z* 430.9.1/268.0 (DP = 76 V, CE = 17 V, CXP = 12 V) for D-xylose, Q1/Q3 of *m*/*z* 430.9.1/268.0 (DP = 71 V, CE = 29 V, CXP = 11 V) for D-ribose, and Q1/Q3 of *m*/*z* 430.9.1/268.0 (DP = 76 V, CE = 17 V, CXP = 9 V) for d-apiose. Chromatography was performed using a Phenomenex Kinetex F5 column (100 × 2.1 mm i.d., 100 Å, 1.7 μm, Phenomenex, Aschaffenburg, Germany) kept at 40 °C. Compound elution was performed with a flow rate of 0.4 mL/min, and the mobile phase contained (A) 1% aqueous formic acid and (B) ACN (1% formic acid) with the following gradient: 0 min, 5% B; at 3 min, 5% B; at 5 min, 20% B; at 25 min, 25% B; at 27 min, 100% B; at 30 min, 100% B; at 31 min, 5% B; at 35 min, 5% B. A comparison of the retention times and mass transitions of reference compounds allowed the identification of the monosaccharides D-glucose and D-apiose, present in the isolated bitter compound **15** from fraction F1-4-12-5.

### Sensory Analysis

#### Sensory Panel Training and Sample Preparation

The 12 panelists (six females and six males, 22–30 years of age) participated in the sensory tests and provided informed consent to participate in the present study. The trained panelists had no history of known taste disorders. They were familiar with the sensory analysis methodologies used and were able to evaluate various chemosensory attributes. Panelists underwent weekly training sessions for at least two years to become proficient in taste terminology and sensory evaluation techniques. Sensory training utilized aqueous reference solutions (2.0 mL, pH 5.9), including sucrose (50 mmol/L) for sweet, L-lactic acid (20 mmol/L) for sour, NaCl (20 mmol/L) for salty, caffeine (1 mmol/L) for bitter, and monosodium l-glutamate (3 mmol/L) for umami taste perception.^18^ All sensory analyses were performed at 22–25 °C in a sensory panel room using the sip-and-spit method. Nose clips were used during all sensory analyses to avoid cross-model interactions with odor-active compounds.

#### Taste Profile Analysis

An aliquot (6 g) of sunflower press cake was suspended in water (100 mL, pH 5.9) and presented to the trained panel. To prevent sedimentation, the suspension was stirred during the sensory test. The trained panelists were asked to evaluate the taste attributes sweet, bitter, umami, salty, astringent, and sour on a scale from 0 (not detectable) to 5 (strongly detectable). Additionally, an aliquot of fraction F1 and subfractions F1-4 was dissolved in bottled water (25 mL, pH 5.9) in natural concentrations and evaluated by the trained sensory panelists regarding bitterness, sweetness, sourness, saltiness, umami, and astringency.

#### Taste Dilution Analysis

The HPLC subfractions F1-4-1–F1-4-17, isolated from an aliquot (320 mg) of fraction F1-4, were dissolved in bottled water (20 mL, pH 5.9) and sequentially diluted 1:1 (v/v) with bottled water. The dilution series was presented to the panel in ascending concentrations, and the taste dilution (TD) factor for bitterness was determined by asking the sensory panel to mark the first detectable difference between the sample and the control (bottled water, pH 5.9).

#### Human Taste Recognition Thresholds

The two-alternative forced choice test was used to determine threshold concentration, at which the bitter taste quality of the compound was just detectable. For this purpose, the purified substances were dissolved in bottled water at increasing concentrations. The individual recognition thresholds were determined by calculating the geometric mean of the first falsely and the last correctly identified concentrations. The taste threshold for the sensory panel was estimated by averaging the threshold values obtained from each panelist.

### High-Performance Liquid Chromatography

The HPLC setup (Jasco, Groß-Umstadt, Germany) consisted of a binary pump system PU-2087 Plus, a DG-4400 degasser, and a Rheodyne injection valve, model Rh 2807i type (Rheodyne, Bensheim, Germany). The effluent was monitored using an MD-2010 Plus diode array detector (Jasco, Groß-Umstadt, Germany) operating within a wavelength range of 200–500 nm, along with a Sedex LT-ELSD detector Model 80 (Sedere, Alfortville, France). Chromatographic separation was performed on a preparative Nucleodur C18 Pyramid column (250 × 21 mm, 5 μm, 80 Å, Macherey-Nagel, Düren, Germany), a semipreparative Luna Phenyl-Hexyl column (250 × 10 mm, 5 μm, 100 Å, Phenomenex, Aschaffenburg, Germany), and Luna Phenyl-Hexyl column (250 × 4.60 mm, 5 μm, 100 Å, Phenomenex, Aschaffenburg, Germany) all equipped with a guard column of the same type. Data acquisition was managed using Galaxie Chromatography Software, version 1.10.0.5590.

### Ultrahigh Performance Liquid Chromatography/Time-of-Flight Mass Spectrometry

High-resolution mass spectra were obtained by injecting 2 μL aliquots of all analytes in ACN/H_2_O (80:20, v/v) into an Acquity UPLC core system (Waters, Manchester, UK). This system included a binary solvent manager, a sample manager, and a column oven. Chromatographic separation was performed on a BEH C18 column (150 × 2.1 mm, 1.7 μm, 130 Å; Waters, Manchester, UK) at a flow rate of 0.4 mL/min and a temperature of 40 °C with 0.1% formic acid in H_2_O (v/v) as solvent A and 0.1% formic acid in ACN (v/v) as solvent B. For the initial screening of fractions, the gradient started at 5% B and increased to 100% B within 8 min and remained isocratic for 5 min. The methods used were previously reported in the literature.^18,22^ High-resolution mass spectra were acquired on a Synapt G2-S HDMS (Waters, Manchester, UK) in positive and negative ESI resolution modes using a capillary voltage of 2.5 kV and −1.7 kV, respectively; 50 V sampling cone; 4.0 kV extraction cone; 150 °C source temperature; 450 °C desolvation temperature, 2 and 30 L/h cone gas, and 800 L/h desolvation gas. The mass spectrometer was calibrated across a range of *m*/*z* 50–1200 using a sodium formate solution (0.5 mmol/L) in isopropanol/H_2_O (90:10, v/v). The data were lock mass corrected by infusing a solution of leucine enkephalin (1 ng/μL, *m*/*z* 556.2771, [M + H]^+^ and *m*/*z* 554.2615, [M – H]^−^) at 10 μL/min. Data processing was performed using MassLynx 4.2 (Waters, Manchester, UK).

### Quantification of Fatty Acids and Fatty Acid Oxidation Products Using Liquid Chromatography–Differential Mobility Separation–Tandem Mass Spectrometry

The reference compounds were obtained commercially, and to ensure accurate quantification, two different commercially available internal standards structurally similar to the analytes were selected: [^13^C_18_]-linoleic acid (IS 1) was used as the internal standard for fatty acids, whereas 18-hydroxyoleic acid (IS 2) served as an internal standard for the oxylipins. MS/MS parameters for each analyte and internal standard were optimized individually in ESI negative ionization mode to monitor the fragmentation of pseudomolecular ions.^25^

### Solvent Extraction for Quantification

To perform the quantification in triplicate, 3 × 500 mg of sunflower press cake, a mixture of MeOH/H_2_O (50:50, v/v, 5 mL), and the following internal standard solutions were added to a cryogenic tube (10 mL, VWR Chemicals, Fontenay-sous-Bois, France): 25 μL of [^13^C_18_]-linoleic acid (IS1, 0.5 mM in MeOH) and 25 μL of 18-hydroxyoleic acid (IS 2, 0.5 mM in MeOH). The extraction was performed using an Analogue Orbital Shaker 3005 (GFL, Burgwedel, Germany) for 1 h at 300 U/min. The extracts were membrane filtered (Minisart RC 15, 0.45 μm, Sartorius AG, Göttingen, Germany) and subsequently injected into the liquid chromatography–differential mobility separation–tandem mass spectrometry (LC–DMS–MS/MS) system.^25^

### Calibration Curve

The exact concentration of the analytes was verified using quantitative NMR (qNMR), and a stock solution (0.2 mM) was prepared in MeOH. This stock solution was diluted to 0.1, 0.05, 0.025, 0.0125, 0.0063, 0.0031, 0.0016, 0.0008, and 0.0004 mM. Next, 1 mL of each dilution was mixed with 10.1 μL of an internal standard solution mixture. The UHPLC–DMS–MS/MS analysis of each sample was performed in triplicate. Then, calibration curves were prepared by plotting the peak area ratio of the analyte to the internal standard versus the concentration ratio of each analyte to the internal standard. Linear regression was used for quantitation using MultiQuant version 3.03 (Sciex, Darmstadt, Germany).^22^

### Ultrahigh Performance Liquid Chromatography–Differential Mobility Separation–Tandem Mass Spectrometry System and Parameters

The MS/MS analysis was performed on a QTrap 6500+ mass spectrometer equipped with a SelexION + DMS cell (Sciex, Darmstadt, Germany) in the negative ionization mode, as reported in the literature.^25^ The ion mobility parameters were as follows: isopropanol as the chemical modifier at a flow rate of 363.6 μL/min (low), a separation voltage (SV) of 3500 V, a DMS temperature of 225 °C (medium), and a DMS offset of 3 V. The declustering potential (DP), entrance potential (EP), collision energy (CE), and cell exit potential (CXP) were optimized using commercial references of methanolic solutions of the analytes and internal standards.^25^

The mass spectrometer was operated in the MRM full scan mode (ion spray voltage: −4500 V for ESI negative ionization) with the following parameters: temperature, 450 °C; gas 1, 55 psi; gas 2, 65 psi. The MS/MS system was coupled to a Shimadzu LC system Nexera X3 (Shimadzu, Duisburg, Germany) consisting of a Shimadzu LC-40D pump, a Shimadzu DGU-405 degasser, a Shimadzu SIL-40C autosampler, a Shimadzu CTO-40C column oven AC, and a Shimadzu SCL-40 control unit.

Sample injections (1 μL) were followed by chromatography on a Kinetex C18, (150 × 10 mm, 1.7 μm; Phenomenex, Aschaffenburg, Germany) with a binary gradient using 5 mM NH_4_Ac in H_2_O (pH 5) as solvent A and 5 mM NH_4_Ac in H_2_O (pH 5)/ACN/isopropanol (5:55:40, v/v/v) as solvent B (flow rate of 0.35 mL/min): 0 min, 15% B; 0.5 min, 15% B; 2 min, 30% B; 6 min, 50% B; 17 min, 71% B; 19 min, 100% B; 21 min, 100% B; 22 min, 15% B; 24 min, 15% B. The instrument was controlled using the Analyst 1.6.3 software (Sciex, Darmstadt, Germany). Data analysis was performed using Microsoft Excel (Microsoft Office, 2016) and Multiquant (version 3.0.3, Sciex, Darmstadt, Germany).

### Quantification of Bitter Compound **15** Using Ultrahigh Performance Liquid Chromatography–Tandem Mass Spectrometry. Solvent Extraction for Quantification

The sunflower press cake (1 g, n = 3) was weighed into bead beater tubes (15 mL, CKMix, Bertin Technologies, Montigny-le-Bretonneux, France), and a mixture of methanol and water (50:50, v/v, 5 mL) was added. Extractive grinding was performed at 6000 rpm for 3 × 30 s with 30 s breaks in between using a bead beater (Precellys Homogenizer, Bertin Technologies, Montigny-le-Bretonneux, France). The samples were centrifuged (10 min, 4800 × g), and the clear supernatant was separated. The residue was extracted using the same protocol a total of five times prior to quantification to assess the viability of external calibration. Therefore, the individual extraction steps were injected into the LC-MS system and the compound areas were compared. After three extractions, less than 5% of the initial compound area was detected, which remained consistent after two additional extractions (Table S1). In total, five extraction steps were deemed sufficient for quantification. The combined supernatants of all five extraction steps were evaporated to dryness under nitrogen and reconstituted in a mixture of methanol and water (50:50, v/v, 400 μL), transferred to autosampler vials, and stored at −20 °C until LC-MS analysis.

### Ultrahigh Performance Liquid Chromatography–Tandem Mass Spectrometry System and Parameters

Mass spectrometry was conducted using the QTRAP 6500 system described earlier, operated in ESI negative mode with the following ion source parameters: ion spray voltage at −5500 V (ESI^–^), curtain gas at 35 psi, nebulizer gas at 55 psi, heater gas at 65 psi, collision-activated dissociation medium, and source temperature at 500 °C. The MS system was coupled to the Shimadzu Nexera X2 UHPLC (Shimadzu, Duisburg, Germany) mentioned earlier. The MS/MS parameters of compound **15** were tuned and optimized, resulting in the characteristic Q1/Q3 transitions of *m*/*z* 607.2/161.0 (DP = −140 V, CE = −46 V, CXP = −17 V) as the quantifier, and *m*/*z* 607.2/178.9 (DP = −140 V, CE = −44 V, CXP = −21 V) as the qualifier. Chromatography was performed using a Kinetex Biphenyl column (100 × 2.1 mm, 1.7 μm, 100 Å, Phenomenex, Aschaffenburg, Germany) maintained at 40 °C. Aliquots (1 μL) were injected into the system at a flow rate of 0.4 mL/min and using 0.1% formic acid in water and 0.1% formic acid in methanol as solvents A and B, respectively, with the following gradient: 5% B held for 1 min, increased in 2.5 min to 60% B, held at 60% B for 2 min, increased in 1 min to 100% B, and held at 100% for 1 min, then decreased in 1.5 min to 5% B, and re-equilibrated for 1.5 min at 5% B.

### Calibration Curve

For quantification, a stock solution of bitter tastant **15** was prepared in a mixture of methanol and water (50:50, v/v). The concentration was determined using quantitative ^1^H NMR spectroscopy. The stock solution (10.96 mmol/L) was then diluted successively (1:2, 1:4, 1:8, 1:16, 1:32, 1:64, 1:128, and 1:256), using the same solvent mixture. All dilutions were analyzed by means of UHPLC-MS/MS using a scheduled (20 s window) multiple reaction monitoring (MRM) method. Then, the peak area was plotted against the concentrations, and an external calibration curve was established with linear regression (y = 2667.8x + 386458, R^2^ = 0.9992), which was used for quantification of **15** in the sunflower press cake.

### Nuclear Magnetic Resonance Spectroscopy

NMR spectra were recorded using a Bruker Avance Neo 600 MHz system (Bruker, Rheinstetten, Germany) equipped with a cryo-TCI probe at 300 K. The samples were prepared using 100 × 3 mm NMR tubes (Hilgenberg, Münnerstadt, Germany). The data acquisition and processing were performed using TopSpin 4.1.1 (Bruker, Rheinstetten, Germany) and MestReNova 11.0.4 (Mestrelab Research, La Coruña, Spain). Chemical shifts were referenced to the residual solvent signals of DMSO-*d*_6_ or D_2_O.

#### Quantitative NMR spectroscopy (qHNMR)

For quantification, data were recorded on a Bruker AV III 400 MHz system, which was equipped with a Broadband Observe BBFOplus probe. The concentration of the target compounds was determined with the external reference l-tyrosine (5.21 mmol/L) via the ERETIC II procedure, as described by Frank et al. (2014).^26^

## Results and Discussion

This study aimed to identify and characterize key bitter compounds in sunflower press cake that have not yet been extensively researched in terms of their taste profile. The results offer novel insights into the sensory properties of sunflower press cake, thereby presenting opportunities to enhance its palatability and broaden its applicability within the food industry.

### Sensory Analysis and Sequential Solvent Extraction of Sunflower Press Cake

The sunflower press cake was first analyzed by a trained panel by applying a taste profile analysis to gain initial insight. On a scale from 0 (not detectable) to 5 (strongly detectable), the panelists were asked to rank the taste intensity of bitter, sweet, sour, umami, salty, and astringent. Bitterness and astringency exhibited the highest intensity ratings, with scores of 2.5 and 2.4, respectively, followed by sourness with a score of 0.8. In comparison, sweetness and saltiness were perceived with lower intensity, each scoring 0.4, whereas umami was perceived with the lowest intensity score of 0.3. Based on the prominent bitterness detected during the sensory analysis, the sensomics approach was subsequently applied to identify the key bitter compounds responsible for the off-taste in sunflower press cake. Therefore, sunflower press cake was extracted sequentially with a series of solvents, beginning with MeOH/H_2_O (F1), followed by MeOH (F2), ethyl acetate (F3), and *n*-pentane (F4). A rotary evaporator and freeze-dryer were used to remove the solvent from each fraction. Subsequently, fractions were dissolved in their natural concentrations in water and analyzed using comparative taste profile analysis (Table 1). Fraction F1, with an intensity score of 2.5, showed the highest bitterness compared with fractions F2–F4. Due to its high bitterness intensity, fraction F1 was further fractionated to identify the key bitter molecules.

**Table 1:** Sensory Evaluation of Sunflower Press Cake Isolated Fractions^a^

Taste Intensities for Individual Fractions^a^					
Taste attributes	Press cake	F1	F2	F3	F4
sweet	0.4	0.3	0.1	0.2	0.2
sour	0.8	0.6	0.2	0.5	0.3
umami	0.3	0.2	0.1	0.2	0.1
salty	0.4	0.2	0.1	0.2	0.3
bitter	2.5	2.5	1.0	1.1	0.9
astringent	2.4	1.8	1.0	1.1	1.1

a The panelists were asked to rate aqueous solutions of the natural concentrations of the fractions F1–F4 and the residue.

b The intensity of the individual taste descriptors was rated by a trained panel on a scale from 0 (not detectable) to 5 (strongly detectable).

### Activity-Guided Identification of the Key Bitter Compounds in Fraction F1

To identify the fraction with key bitter compounds, F1 was separated further with RP-18 solid-phase extraction into five subfractions (F1-1 to F1-5). Fractions F1-1–F1-5 were separated from the solvent and used for sensory analysis. Fraction F1-4 showed higher bitterness (3.0) than other subfractions (Figure 1). Fraction F1-4 was further separated using preparative RP18-HPLC, and 17 subfractions were collected (F1-4-1 to F1-4-17). Subfractions were separated from the solvent and dissolved in equal amounts of water for taste dilution analysis at ascending concentrations. According to the TD factor analysis of 17 subfractions, fractions F1-4-12, F1-4-15, and F1-4-16 showed high bitterness with TD factors of 32, 28, and 32, respectively (Figure 2). Following this analysis, the subfractions were screened using UPLC-TOF-MS to determine their compound complexity and to assess whether further subfractionation was necessary. This screening suggested that the bitter fractions F1-4-15 and F1-4-16 may contain fatty acids and fatty acid oxidation products; therefore, we focused on the fatty acid components in subsequent analyses. These compounds were further characterized using LC-TOF-MS (ESI^–^) analysis, which revealed pseudomolecular ions ([M – H]^−^) with *m*/*z* values of 329.2329, 329.2330, 329.2332, 295.2277, 295.2274, and 293.2113. On the basis of the elution times of those fractions and fragmentation patterns, it was hypothesized that these compounds are lipid oxidation products previously identified in pea protein and poppy seeds, which are known to cause bitterness.^18,22^ Furthermore, known taste-active compounds and free fatty acids were screened and analyzed against reference standards using LC-MS/MS and UPLC-TOF-MS. The results indicated that trihydroxy­octadecenoic acids and hydroxy­octadecenoic acids, found in various plant-based products, were also present in sunflower press cake.

**Figure 1:**
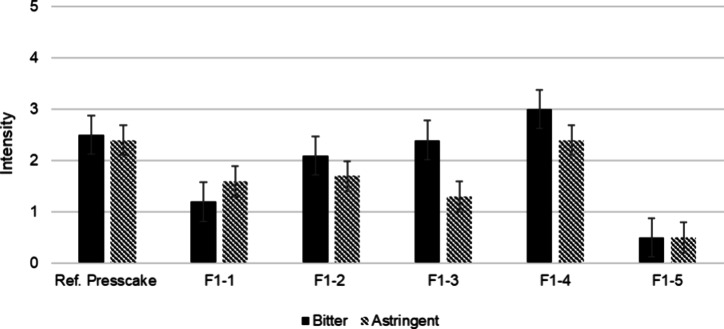
Sensory analysis of solid-phase extraction fractions F1-1–F1-5 isolated from sunflower press cake. The panelists were asked to rate the bitterness and astringency on a scale from 0 (not detectable) to 5 (strongly detectable). Error bars represent the 95% confidence interval of the mean value.

**Figure 2:**
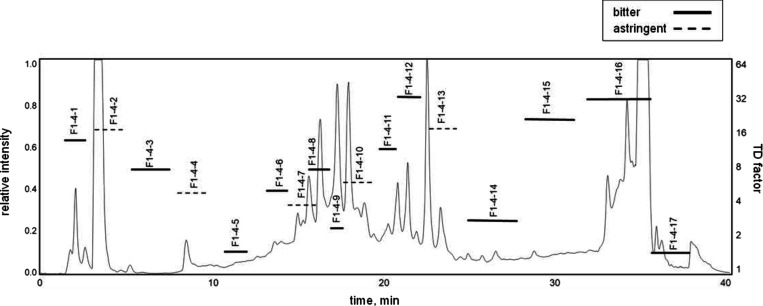
Reversed-phase high-performance liquid chromatography with evaporative light scattering detection (RP-HPLC ELSD) chromatogram of F1-4 along with taste dilution (TD) factors of collected subfractions F1-4-1–F1-4-17.

The identified trihydroxyoctadecenoic acids included 9,12,13-trihydroxyoctadec-10-enoic acid (**1**) (*m*/*z* 329.2329, [C_18_H_33_O_5_]^−^), 9,10,11-trihydroxyoctadec-12-enoic acid (**2**) (*m*/*z* 329.2330, [C_18_H_33_O_5_]^−^), and 11,12,13-trihydroxyoctadec-9-enoic acid (**3**) (*m*/*z* 329.2332, [C_18_H_33_O_5_]^−^). Further lipid oxidation derivatives including (10*E*,12*E*)-9-hydroxyoctadeca-10,12-dienoic acid (**4**) (*m*/*z* 295.2277, [C_18_H_31_O_3_]^−^), (10*E*,12*Z*)-9-hydroxyoctadeca-10,12-dienoic acid (**5**) (*m*/*z* 295.2273, [C_18_H_31_O_3_]^−^), (9*E*,11*E*)-13-hydroxyoctadeca-9,11-dienoic acid (**6**) (*m*/*z* 295.2274, [C_18_H_31_O_3_]^−^), (9*Z*,11*E*)-13-hydroxyoctadeca-9,11-dienoic acid (**7**) (*m*/*z* 295.2271, [C_18_H_31_O_3_]^−^) and (9*Z*,11*E*)-13-oxooctadeca-9,11-dienoic acid (**8**) (*m*/*z* 293.2113, [C_18_H_29_O_3_]^−^) was detected.

Free fatty acids affecting to the flavor profile of sunflower press cake included α-linolenic acid (**9**) (*m*/*z* 277.2180, [C_18_H_29_O_2_]^−^), linoleic acid (**10**) (*m*/*z* 279.2336, [C_18_H_31_O_2_]^−^), oleic acid (**11**) (*m*/*z* 281.2479, [C_18_H_33_O_2_]^−^), palmitic acid (**13**) (*m*/*z* 255.2324, [C_16_H_31_O_2_]^−^), and stearic acid (**14**) (*m*/*z* 283.2630, [C_18_H_35_O_2_]^−^). These compounds and 2-hydroxyoleic acid (**12**) (*m*/*z* 297.2433, [C_18_H_33_O_3_]^−^) are well-documented contributors to the sensory characteristics of some plant-based matrices, particularly bitterness and fatty taste attributes (Figure 3).

**Figure 3:**
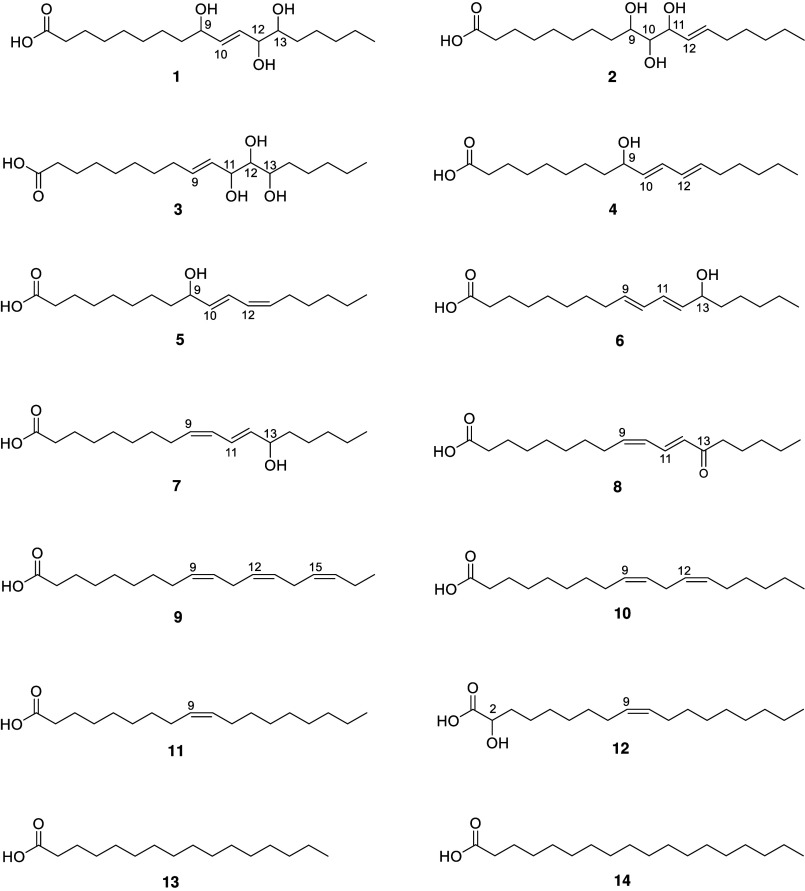
Chemical structures of identified compounds from sunflower press cake: 9,12,13-trihydroxyoctadec-10-enoic acid (**1**), 9,10,11-trihydroxyoctadec-12-enoic (**2**), 11,12,13-trihydroxyoctadec-9-enoic acid (**3**), (10*E*,12*E*)-9-hydroxyoctadeca-10,12-dienoic acid (**4**), (10*E*,12*Z*)-9-hydroxyoctadeca-10,12-dienoic acid (**5**), (9*E*,11*E*)-13-hydroxyoctadeca-9,11-dienoic acid (**6**), (9*Z*,11*E*)-13-hydroxyoctadeca-9,11-dienoic acid (**7**), (9*Z*,11*E*)-13-oxooctadeca-9,11-dienoic acid (**8**), α-linolenic acid (**9**), linoleic acid (**10**), oleic acid (**11**), 2-hydroxyoleic acid (**12**), palmitic acid (**13**), stearic acid (**14**), and pinocarveol β-d-apiofuranosyl-(1→6)-β-d-(4-*O*-caffeoyl) glucopyranoside (**15**) (shown in Figure 4).

The E/Z isomer configurations were identified by comparing the retention times of the analytes to those of corresponding commercial reference compounds using LC-TOF-MS. Hydroxyoctadecadienoic acids (**4**–**7**) are recognized as metabolites in the lipoxygenase pathway, produced through the enzymatic activity of 9-/13-LOX and subsequent reduction by peroxygenases.^27–29^ Their bitter activity has been previously reported.^18^ Similarly, trihydroxyoctadecenoic acids (**1**–**3**) have been previously identified in various plants and plant-based products, as well as their bitter activity.^18,22^ The 2-hydroxy derivatives of the α-oxidation enzyme system of other plants^29–31^ and the bitter taste threshold has been identified.^22^ The (9*Z*,11*E*)-13-oxooctadeca-9,11-dienoic acid was previously identified as a product of the enzymatic pathway, where hydroperoxides are produced by 9- and 13-LOXs, followed by dehydration or dehydrogenation of fatty acid hydroxides.^22^

Fraction F1-4-12, displayed a significantly more complex composition, requiring further fractionation. Therefore, it was further fractionated by semipreparative HPLC, and eight fractions were collected. The bitter target compound was contained in fraction F1-4-12-5, which was further purified with analytical HPLC for final structural analysis. After the isolation of the target compound by iterative HPLC fractionation, the structure of **15** (Figure 4) was determined using TOF-MS and 1D-/2D-NMR. First, high-resolution mass spectra were acquired, which showed a mass-to-charge (*m*/*z*) ratio of 607.2404, resulting in a predicted elemental composition of C_30_H_39_O_13_ ([M – H]^−^) in ESI negative mode. The calculated *m*/*z* ratio (607.2396 *m*/*z* for C_30_H_39_O_13_, [M – H]^−^) was in good agreement with the measured value, indicated by a mass error of 1.58 ppm. Additionally, MS^e^ spectra showed characteristic fragment ions, previously reported^32^ with *m*/*z* ratios of 179.0348, 161.0240, and 135.0444, all of which suggested that caffeic acid (179.0349 *m*/*z*, [M – H]^−^) is a putative constituent of **15** (Figure 5, **A**). To further elucidate the structure of **15**, NMR experiments were performed (Table 2). The ^1^H and ^13^C NMR spectra of compound **15** exhibited signals typical of a trans-caffeoyl moiety, which was identified via the AMX spin system consisting of the proton resonances H–C(9′), H–C(8′), and H–C(5′) at 6.76, 6.98, and 7.05 ppm. The characteristic coupling pattern as well as the coupling constants of the aromatic signals—8.2 Hz (doublet), 8.2/1.9 Hz (doublet of doublets), and 1.9 Hz (doublet)—were in agreement with the values reported in the literature.^33^ The distinctive trans-olefinic proton signals resonating at 6.26 and 7.47 ppm, with a coupling constant of 15.8 Hz (doublet), supported by nine carbon signals C-1′–C-9′ (δ 166.1, 114.2, 146.2, 124.7, 114.8, 145.9, 150.8, 122.2, 116.1) in the ^13^C NMR spectrum, confirmed the presence of caffeic acid as a substructure of **15** (Figure 5, B). In addition, the ^1^H NMR spectrum displayed eight proton signals in the aliphatic region, integrating for 12 protons. The signals resonating at 0.59 and 1.21 ppm could be assigned to methyl groups (H-(9), H–C(10)) integrating for three protons each. Two diastereotopic methylene groups resonating at 1.92–1.95 ppm/2.05–1.14 ppm (H–C(2)) and 1.58/2.24–2.30 ppm (H–C(8)) as well as three methine protons at 1.88–1.93 ppm (H–C(3)), 2.41 ppm (H–C(5)), and 4.39 ppm (H–C(1)) were assigned using the heteronuclear (C,H) single quantum coherence and homonuclear (H,H) correlated spectroscopy experiments. Moreover, the presence of an exocyclic double bond, resonating at 4.81/5.01 ppm (H–C(7)), was assigned using heteronuclear multiple bond correlation (HMBC) spectroscopy, optimized for ^2^ *J*_C,H_ and ^3^ *J*_C,H_ couplings. The correlation of proton signals H–C(1), H–C(3), H–C(5), H–C(7), H–C(8), H–C(9), and H–C(10) with carbon C-5 (50.4 ppm) cumulatively indicated the presence of a pinocarveol moiety. This assignment was supported by the identification of the two quaternary carbon atoms C-4 at 40.1 ppm and C-6 at 151.2 ppm, which were determined using ^13^C NMR. The presence of a pinocarveol group was further supported by the respective carbon signals C-1–C-7 (δ 72.6, 32.2, 39.6, 40.1, 50.4, 151.2, 114.1, 27.2, 1.2, 0.6), which closely resembled previously reported values^34^ (Figure 5C). In addition to caffeic acid and pinocarveol, the presence of two carbohydrate moieties in **15** was indicated by the presence of two anomeric proton signals at 4.37 ppm (H–C(1̀̀′′)) and 4.79 ppm (H–C(1̀̀̀′′′)). It is hypothesized that these residues include a hexose and a branched-chain pentose, evidenced by the ^13^C NMR spectrum, which showed 11 aliphatic carbon signals, including one quaternary carbon (C-3̀̀̀′′′) at 79.6 ppm and three methylene carbon resonance signals at 67.4, 63.6, and 73.8 ppm, respectively. The carbohydrates were ultimately identified as d-glucose and D-apiose after acidic hydrolysis, followed by chemical derivatization with l-cysteine methyl ester and phenyl isothiocyanate using LC-MS/MS, as described previously.^35^ The β-glycosidic linkage of the glucose was indicated by the coupling constant of 7.9 Hz of H–C(1̀̀′), and the anomeric configuration of the apiose moiety was determined to be β on the basis of a comparison of the ^13^C NMR data for **15** with those of α- and β-d-apiofuranoside^36^ and coupling constants of 2.8 Hz (H–C(1̀̀̀′′′)) consistent with the reported data for β-D-apiofuranoside (*J* = 2.6 Hz).^37–39^ Finally, the connections of the individual substructures were determined via the HMBC correlations of H–C(1) of pinocarveol (4.39 ppm) with C-1̀̀ (101.8 ppm) of glucose, the proton at position H–C(4̀̀′′) of glucose (4.59 ppm) and C-1̀′ of caffeic acid (169.1 ppm), as well as the methylene protons H–C-(6̀̀′′) of glucose (3.36/3.49 ppm) with C(1̀̀̀′′′) of apiose (109.7 ppm), as highlighted in Figure 5D. Consequently, compound **15** was identified as pinocarveol β-d-apiofuranosyl-(1→6)-β-d-(4-*O*-caffeoyl) glucopyranoside. To the best of our knowledge, compound **15** has not yet been described in the literature. Though similar phenolic glycosides like benzyl alcohol β-d-apiofranosyl-(1→6)-β-d-(4-O-caffeoyl) glucopyranoside were described as potent antioxidants found in sunflower seeds.^38^ Additionally, multiple terpenoid glycosides, like Campholenol-10-O-β-d-apiofuranosyl-(1→6)-β-d-glucopyranoside, Myrtenol-10-O-α-d-apiofuranosyl-(1→6)-β-d-glucopyranoside among others were shown to possess protective effects against H_2_O_2_-induced myocardial cell injury.^39^ However, there are no reports indicating any taste activity.

**Figure 4:**
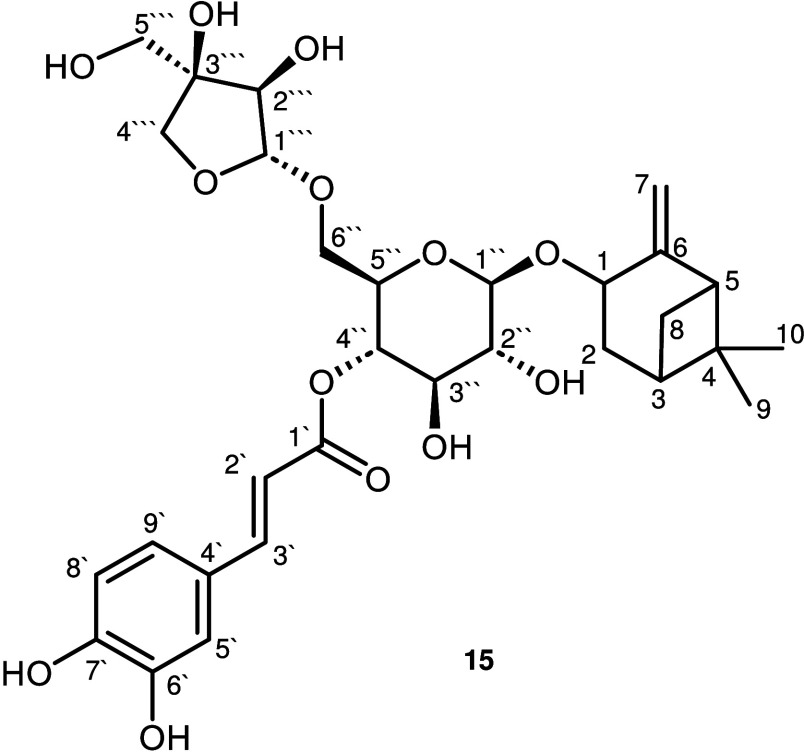
Chemical structure of pinocarveol β-d-apiofuranosyl-(1→6)-β-d-(4-*O*-caffeoyl) glucopyranoside. Arbitrary carbon numbering refers to nuclear magnetic resonance (NMR) assignments given in Table 2.

**Figure 5:**
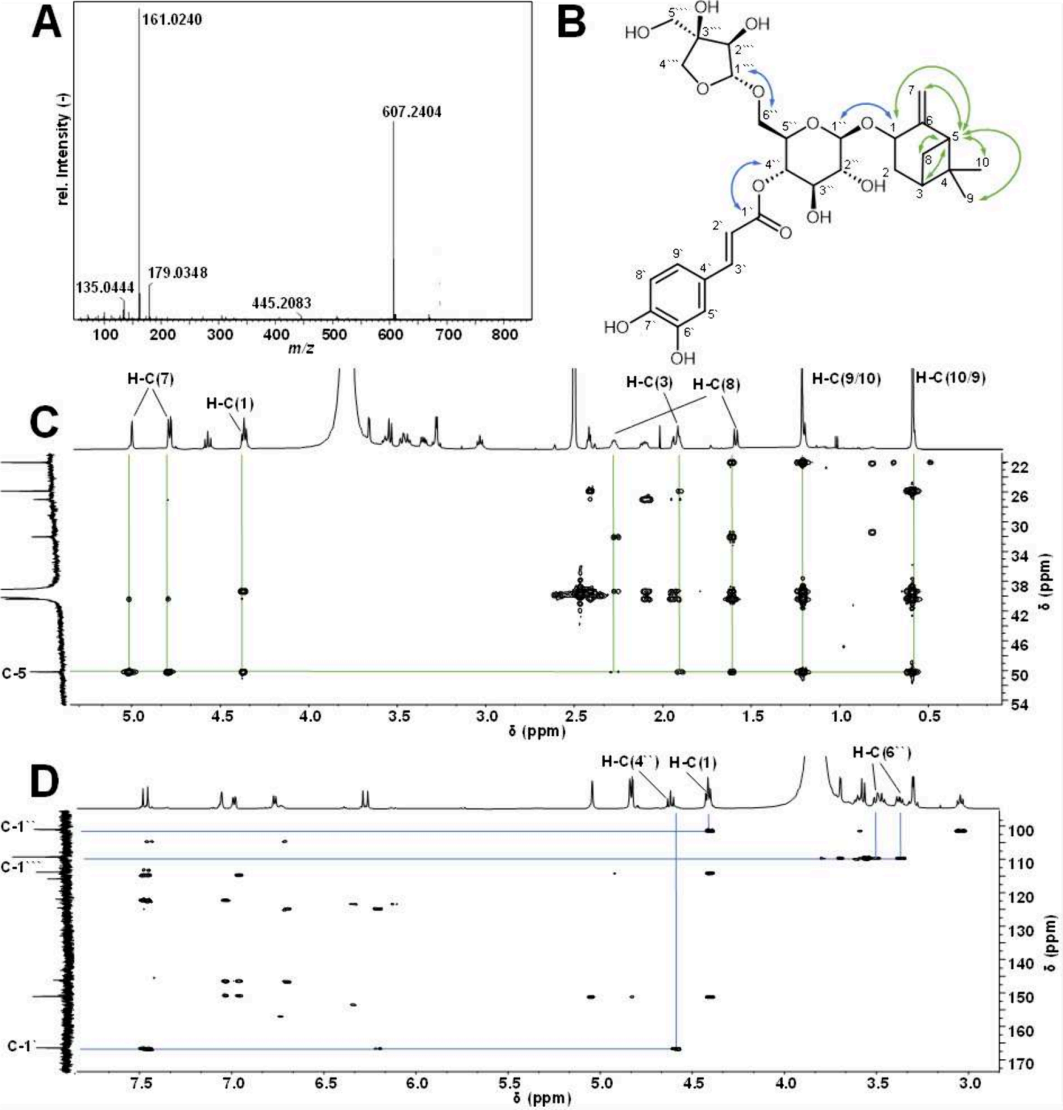
MS^e^ (20–40 eV, ESI^–^ mode) spectrum of bitter tastant **15** isolated from sunflower press cake (A). Chemical structure of **15** showing key correlations for structural elucidation via nuclear magnetic resonance (NMR) spectroscopy (B). Excerpts of heteronuclear multiple bond correlation (HMBC) spectrum (600/150 MHz, DMSO-*d*_6_, 300 K) of **15** indicating the presence of a pinocarveol moiety (C) and the connection of individual substructures (D).

**Table 2:** ^1^H and ^13^C NMR Assignments (600/150 MHz, DMSO-*d*_6_, 300 K) of Pinocarveol d-Apiofuranosyl-(1→6)-β-d-(4-*O*-caffeoyl) Glucopyranoside

	Position	δ_C_ (ppm)	HSQC	δ_H_ (ppm)	M (*J*, Hz)
pinocarveol	1	72.6	[CH]	4.39	d (*J* = 7.3 Hz)
	2	32.2	[CH_2_]	1.92–1.95	m
				2.05–2.14	m
	3	39.6	[CH]	1.88–1.93	m
	4	40.1	[C]	-	-
	5	50.4	[CH]	2.41	t (*J* = 5.4 Hz)
	6	151.2	[C]	-	-
	7	114.1	[CH_2_]	4.81	s
				5.01	s
	8	27.2	[CH_2_]	1.58	d (*J* = 9.5 Hz)
				2.24–2.30	m
	9	1.21	[CH_3_]	25.9	s
	10	0.59	[CH_3_]	22.3	s
caffeic acid	1′	166.1	[C]	-	-
	2′	114.1	[CH]	6.26	d (*J* = 15.8 Hz)
	3′	146.2	[CH]	7.47	d (*J* = 15.8 Hz)
	4′	124.7	[C]	-	-
	5′	114.8	[CH]	7.05	d (*J* = 2.2 Hz)
	6′	145.9	[C]	-	-
	7′	150.8	[C]	-	-
	8′	122.2	[CH]	6.98	dd (*J* = 1.9, 8.3 Hz)
	9′	116.1	[CH]	6.76	d (*J* = 8.2 Hz)
β-d-glucose	1″	101.8	[CH]	4.37	d (*J* = 7.8 Hz)
	2″	73.6	[CH]	3.04	t (*J* = 8.5 Hz)
	3″	74.1	[CH]	3.45	t (*J* = 9.5 Hz)
	4″	72.1	[CH]	4.59	t (*J* = 8.5 Hz)
	5″	73.2	[CH]	3.58–3.62	m
	6″	67.4	[CH_2_]	3.36	dd (*J* = 6.3, 11.6 Hz)
				3.49	dd (*J* = 2.4, 11.6 Hz)
β-d-apiose	1‴	109.7	[CH]	4.79	d (*J* = 2.8 Hz)
	2‴	76.0	[CH]	3.66	d (*J* = 2.5 Hz)
	3‴	79.6	[C]	-	-
	4‴	63.6	[CH_2_]	3.26–3.31	m
	5‴	73.8	[CH_2_]	3.55	d (*J* = 9.5 Hz)
				3.76	d (*J* = 9.5 Hz)

### Sensory Activity of Bitter Compounds

To assess the bitter taste properties of compounds in sunflower press cake, we referred to the taste threshold concentrations reported in recent studies.^18,22^ These thresholds were determined using a 3% aqueous ethanol solution in a two-alternative forced choice test to address the solubility challenges of hydrophobic compounds. The reported thresholds for the compounds listed in Table 3 include a notably low bitter threshold for 2-hydroxyoleic acid and slightly higher thresholds for 9,12,13-trihydroxyoctadec-10-enoic acid and its isomeric counterparts (9,10,11-trihydroxyoctadec-12-enoic acid and 11,12,13-trihydroxyoctadec-9-enoic acid) at 0.13 mmol/L. Previously reported thresholds for free fatty acids, such as linoleic acid, oleic acid, palmitic acid, and stearic acid, indicate that these compounds display relatively higher bitter thresholds than α-linolenic acid. The newly identified pinocarveol β-D-apiofuranosyl-(1→6)-β-D-(4-*O*-caffeoyl) glucopyranoside showed a bitter taste recognition threshold of 0.42 mmol/L.

**Table 3:** Bitter Taste Threshold Concentrations and calculated DoT Factors of Compounds Found in Sunflower press cake

compound no.	compound name	concentrations [mmol/kg]	bitter threshold concentration [mmol/L]	DoT factor
**1**	9,12,13-trihydroxyoctadec-10-enoic acid	0.14 ± 0.01	0.13^b^	1.1
**2**	9,10,11-trihydroxyoctadec-12-enoic acid	0.07 ± 0.01	0.13^b^	0.5
**3**	11,12,13-trihydroxyoctadec-9-enoic acid	0.33 ± 0.07	0.13^b^	2.5
**4**	(10*E*,12*E*)-9-hydroxyoctadeca-10,12-dienoic acid	0.03 ± 0.01	0.35^a^ ^,^ ^b^	0.08
**5**	(10*E*,12*Z*)-9-hydroxyoctadeca-10,12-dienoic acid	0.11 ± 0.01	0.79^a^ ^,^ ^b^	0.1
**6**	(9*E*,11*E*)-13-hydroxyoctadeca-9,11-dienoic acid	0.55 ± 0.15	0.97^a^	0.6
**7**	(9*Z*,11*E*)-13-hydroxyoctadeca-9,11-dienoic acid	0.61 ± 0.03	0.79^a^ ^,^ ^b^	0.8
**8**	(9*Z*,11*E*)-13-oxooctadeca-9,11-dienoic acid	0.65 ± 0.06	0.79^a^	0.8
**9**	α-linolenic acid	2.32 ± 0.40	0.28^a^	8.3
**10**	linoleic acid	52.56 ± 2.03	0.93^a^	56.5
**11**	oleic acid	96.64 ± 4.20	0.98^a^	98.6
**12**	2-hydroxyoleic acid	0.01 ± 0.01	0.06^b^	0.2
**13**	palmitic acid	47.31 ± 2.45	0.81^a^	58.4
**14**	stearic acid	28.36 ± 0.37	0.81^a^	35.0
**15**	pinocarveol β-D-apiofuranosyl-(1→6)-β-D-(4-*O*-caffeoyl) glucopyranoside	1.41 ± 0.10	0.42	3.4

a Taste threshold taken from Lainer et al. (2020).

b Taste thresholds are taken from Gläser et al. (2020).

### Quantitation of Bitter Compounds in Sunflower Press Cake and Calculation of Dose-over-Threshold Factors

The free fatty acids (FFAs) oleic acid, linoleic acid, palmitic acid, stearic acid, and α-linolenic were identified as the predominant compounds in the tested sunflower press cake, with concentrations of 96.64, 52.56, 47.31, 28.36, and 2.32 mmol/kg, respectively. Although no other comparative quantitative data for sunflower press cake are available, this distribution is in line with previous studies reporting that oleic and linoleic acids are consistently found in the highest concentrations among FFAs in various sunflower samples.^40–44^ Traditional sunflower oil, which has been widely cultivated, contains moderate levels of oleic acid (14%–39%) and high levels of linoleic acid, typically over 50%, reaching up to 61% in some cases. However, the fatty acid composition of sunflower oil is highly variable depending on the breeding strategies implemented. For instance, high-oleic sunflower varieties have been bred to contain over 75% oleic acid, and high-stearic–high-oleic sunflower varieties contain approximately 15%–20% stearic acid. Other fatty acids such as palmitic acid generally range between 4.6% and 7% across different sunflower oil types, whereas α-linolenic acid remains very low, typically below 0.1% in most varieties.^40,42–52^ The concentrations of trihydroxy-octadecenoic acids (THOAs) and hydroxy-octadecadienoic acids were significantly lower, ranging from 0.07 to 0.33 mmol/kg and 0.03 to 0.61 mmol/kg, respectively.

In addition to fatty acids and their oxidation products, the second most intense bitter fraction, identified by the TDA, was fraction F1-4-12. This fraction was shown to contain pinocarveol β-d-apiofuranosyl-(1→6)-β-d-(4-*O*-caffeoyl) glucopyranoside (**15**) with a bitter threshold concentration of 0.42 mmol/L. To demonstrate the importance of this compound to explain the overall bitter off-taste of sunflower press cake, UHPLC-MS/MS quantification was performed. The analysis of sunflower press cake revealed a concentration of 1.41 mmol/kg of compound **15**. Since this compound was isolated and described for the first time, there are no reference concentration ranges available in the literature. However, isolating protein from plant sources often leads to an enrichment of secondary plant metabolites. These secondary metabolites frequently possess a bitter off-taste and are typically present in concentrations similar to that of compound **15** in the final protein isolates, as reported in the literature.^20,21^

To assess the bitter taste impact of compounds **1**–**15**, dose-overthreshold (DoT) factors were calculated as the ratio of the taste threshold concentration for each specific tastant.^17,53–56^ The DoT signifies taste relevance, with values above 1 indicating a direct contribution to bitterness. The taste threshold values used in these calculations were previously reported in the literature.^18,22,57^ The calculation of DoT factors revealed that oleic acid (DoT 98.6) had the highest bitter taste impact in the tested sunflower press cake, followed by linoleic acid (DoT 56.5), palmitic acid (DoT 58.4), stearic acid (DoT 35.0), and α-linolenic acid (DoT 8.3). The FFAs had DoT values over 1, indicating that this substance class contributes to the bitterness. Among the oxidized fatty acids, THOAs, particularly 9,12,13-trihydroxyoctadec-10-enoic acid (DoT 1.1) and 11,12,13-trihydroxyoctadec-9-enoic acid (DoT 2.5) exhibited the highest bitter impact.

To date, there has been no quantitative analysis of the key bitter compounds present in sunflower press cake. The high DoT values observed for FFAs such as oleic acid, linoleic acid, palmitic acid, stearic acid, and α-linolenic acid as well as the THOAs 9,12,13-trihydroxyoctadec-10-enoic acid and 11,12,13-trihydroxyoctadec-9-enoic acid are consistent with the significant amount of residual oil in sunflower press cake, ranging from 7% to 16.6% depending on the extraction process.^12,49,58,59^ This residual oil contains free fatty acids and their oxidation products and consequently contributes significantly to the perceived bitterness of sunflower press cake. Additionally, this study identified a novel bitter tastant, representing a previously unrecognized class of bitter compounds. Pinocarveol β-D-apiofuranosyl-(1→6)-β-D-(4-*O*-caffeoyl) glucopyranoside showed a DoT value of 3.4, indicating a direct contribution to the overall bitterness of sunflower press cake. Therefore, it can be concluded that, in addition to fatty acids and their oxidation products, compound **15** contributes to the bitter off-flavor in sunflower press cake. Detailed sensory reconstitution, as well as omission experiments, will be performed in a future study to elucidate the precise contribution of individual constituents to the overall off-flavor profile of sunflower press cake and investigate how these compounds are generated during the food processing of sunflower seeds. It is also important to recognize that the chemical composition may be influenced by both the genetic background of the sunflower cultivar and the processing conditions, as these factors can lead to differences in the types and concentrations of free fatty acids and secondary metabolites, ultimately resulting in variations in their contribution to the overall sensory profile.^4^

In summary, the application of the sensomics approach provided detailed insights into the bitter off-taste profile of sunflower press cake, a promising byproduct for sustainable protein sourcing. By identifying key bitter compounds, including free fatty acids and their oxidation products, as well as a novel terpenoid glycoside, this study has established the primary contributors to the off-flavor challenges that limit the broader acceptance of sunflower press cake in food applications. These findings offer a pathway to mitigating bitterness through targeted processing and formulation strategies, paving the way for sunflower press cake to become a more palatable and viable option in addressing the global protein demand. Strategies such as enzymatic hydrolysis, fermentation, selection of clean protein isolates, and flavor masking or texture adaptation have been proposed to mitigate off-flavors in plant proteins.^9^ Future research should focus on refining processing techniques to reduce these compounds while preserving nutritional value, to further enhance the palatability and consumer acceptance of sunflower press cake.

## Supplementary Material



## Notes

Verification of extraction protocol for quantification of **15**, HPLC chromatograms of fraction F1-4-12 and subfraction F1-4-12-5, MS^e^ spectra of fatty acid oxidation products in bitter fractions F1-4-15 and F1-4-16, and ^1^H and ^13^C NMR spectra of **15** (PDF)
