# Supplementary material for: Characterization of Bitter Off-Taste Stimuli in Sunflower Press Cake Using the Sensomics Approach
Source: J Agric Food Chem. 2025 Sep 4;73(37):23548–59. doi: 10.1021/acs.jafc.5c07283 (PMC12447512; doi:10.1021/acs.jafc.5c07283)
Supplement: Supplementary file 1 [file jf5c07283_si_001.pdf]

Supporting Information

Characterization of Bitter Off-Taste Stimuli in Sunflower Press Cake Using the Sensomics Approach

Lachinkhanim Huseynli[1#], Michael Gigl [2#], Jasmin Müller[3], Christoph Walser[3], Oliver Frank[3], Kristel Vene[1], Corinna Dawid [3,4,5*]

[1] Department of Chemistry and Biotechnology, Tallinn University of Technology, Akadeemia tee 15, 12618 Tallinn, Estonia

[2] Junior Research Group Food Processing and Health, ZIEL Institute for Food and Health, Technical University of Munich, Lise-Meitner-Str. 34, D-85354 Freising, Germany

[3] Chair of Food Chemistry and Molecular Sensory Science, TUM School of Life Sciences, Technical University of Munich, Lise-Meitner-Str. 34, D-85354 Freising, Germany

[4] Professorship for Chemosensory Food Systems, TUM School of Life Sciences, Technical University of Munich, Lise-Meitner-Str. 34, D-85354 Freising, Germany

[5] Leibniz Institute for Food Systems Biology at the Technical University of Munich, Lise-Meitner-Str. 34, D-85354 Freising, Germany

[#] These authors contributed equally to this work

Correspondence: [*]corinna.dawid@tum.de; 00498161712902

Table S1. Comparison of signal areas for the optimization of the extraction protocol for UHPLC-MS/MS quantification of compound 15.

Number of extractions	Signal area of **15**
1-3[a]	9.41E+06
4	4.55E+05
5	4.40E+05

[a] sum of the first three extractions

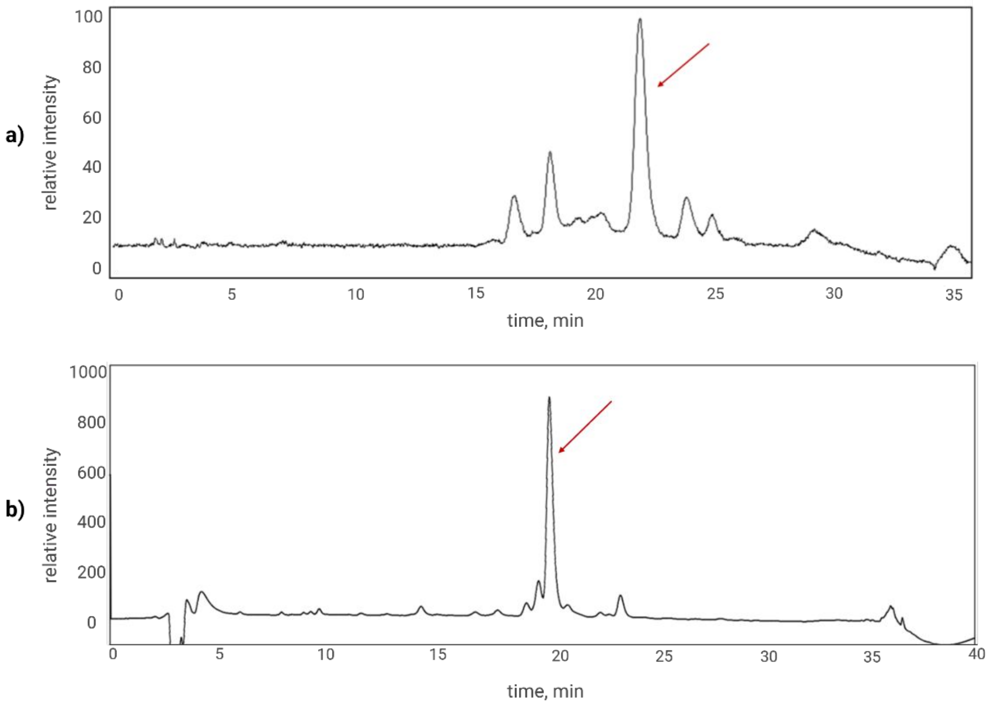

Figure 1. The illustration of the two-step purification approach used for the isolation of the targeted compound. (a) Semi-preparative HPLC chromatogram showing the separation of fraction F1-4-12. The red arrow indicates the peak corresponding to compound F1-4-12-5, (b) Analytical HPLC chromatogram with UV detection of fraction F1-4-12-5, showing improved resolution and a dominant peak corresponding to the targeted compound.

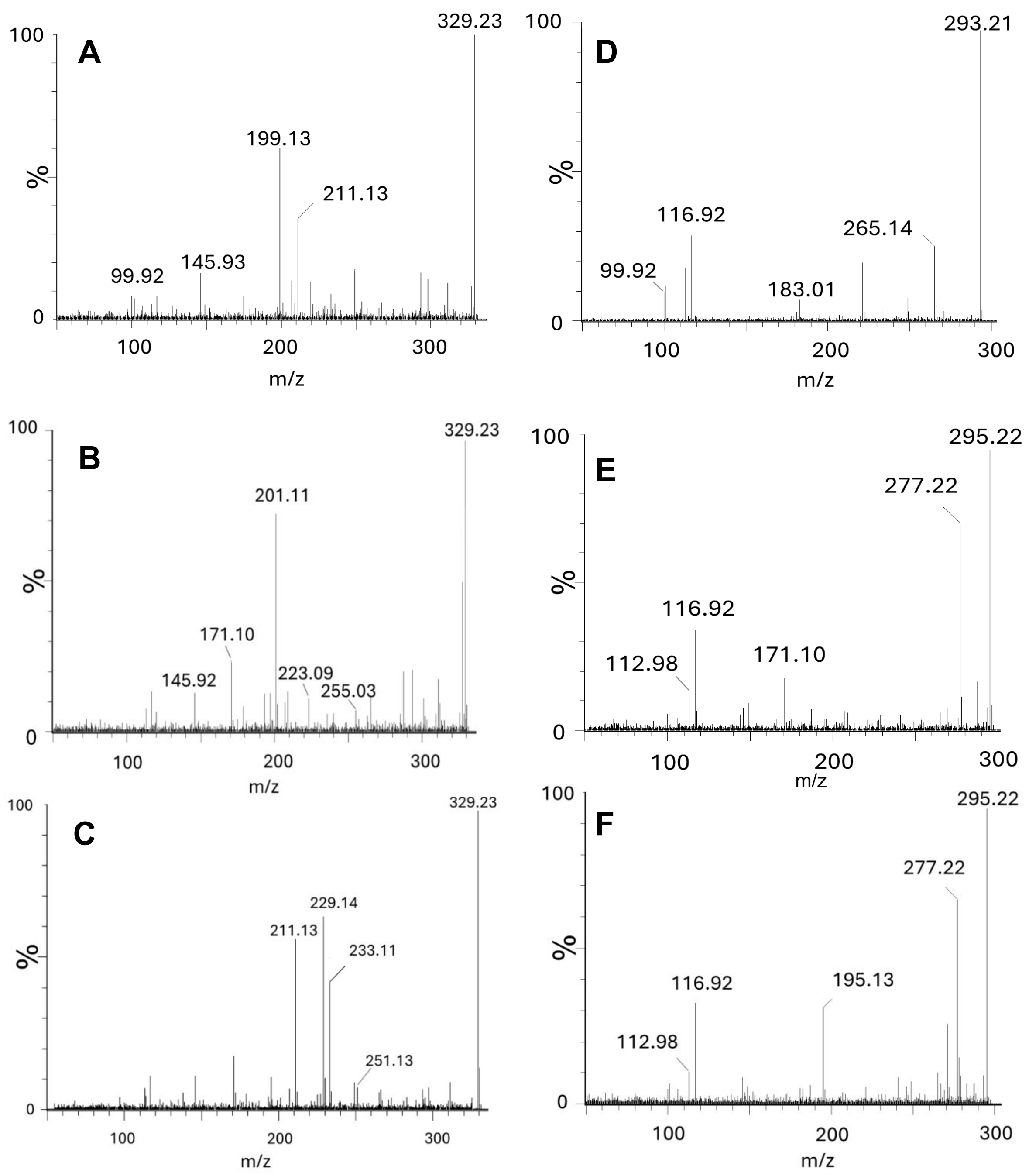

Figure S2. MS^{e} spectra of fatty acid oxidation products found in bitter fraction F1-4-15 and F1-4-16. (**A**) 11,12,13-trihydroxyoctadec-9-enoic acid, (**B**) 9,10,11-trihydroxyoctadec-12-enoic; (**C**) 9,12,13-trihydroxyoctadec-10-enoic acid; (**D**) (9*Z*,11*E*)-13-oxooctadeca-9,11-dienoic acid; (**E**) (10*E*,12*E*)-9-hydroxyoctadeca-10,12-dienoic acid; (**F**) (9*E*,11*E*)-13-hydroxyoctadeca-9,11-dienoic acid

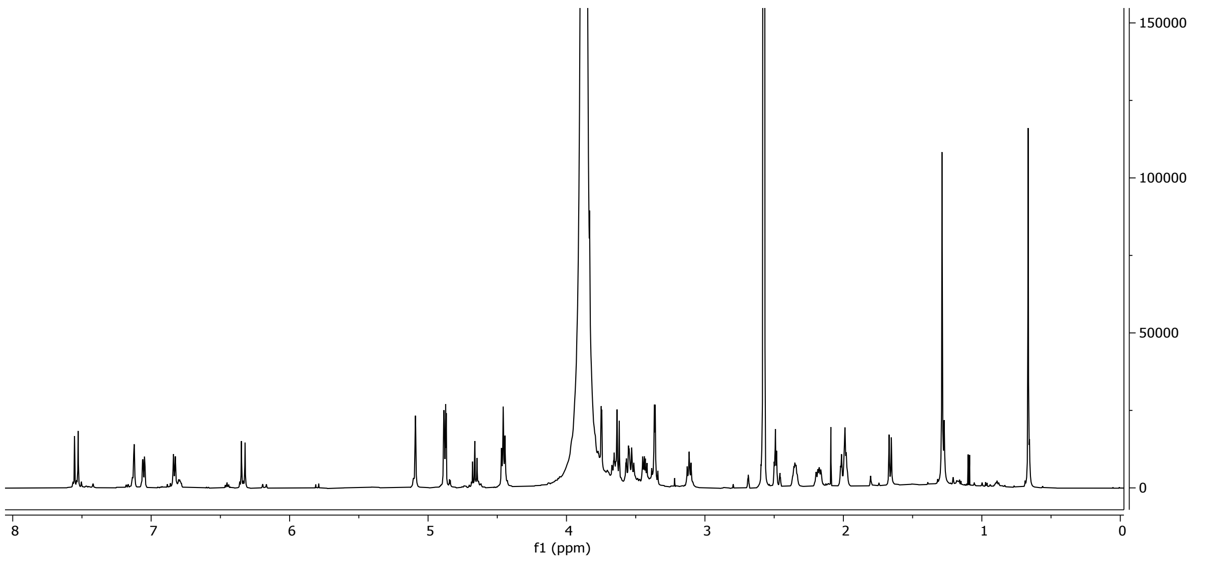

Figure S3. ^{1}H-NMR spectrum of compound **15** (600 MHz, DMSO-d_6, 300K).

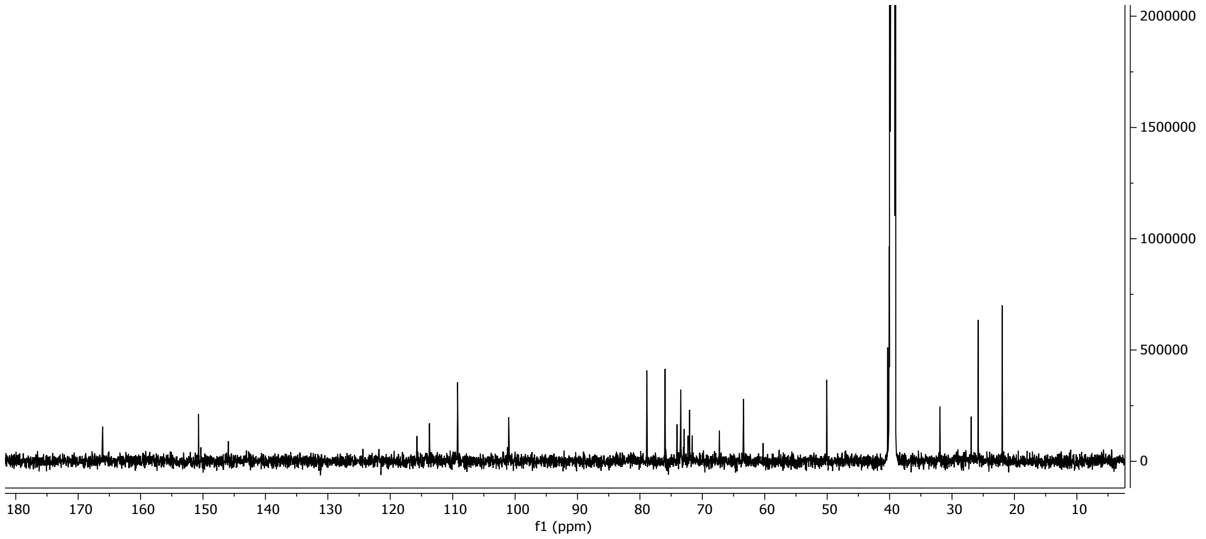

Figure S4. ^{13}C-NMR spectrum of compound **15** (150 MHz, DMSO-d_6, 300K).
